# The protective role of endothelial GLUT1 in ischemic stroke

**DOI:** 10.1002/brb3.3536

**Published:** 2024-05-15

**Authors:** Qiwei Peng, Weiqi Zeng

**Affiliations:** ^1^ Department of Critical Care Medicine, Union Hospital Tongji Medical College, Huazhong University of Science and Technology Wuhan China; ^2^ Key Laboratory of Anesthesiology and Resuscitation (Huazhong University of Science and Technology) Ministry of Education Wuhan China; ^3^ Department of Neurology The First People's Hospital of Foshan Foshan China

**Keywords:** endothelial function, GLUT1, ischemic stroke, neuroprotection

## Abstract

**Objective:**

To provide thorough insight on the protective role of endothelial glucose transporter 1 (GLUT1) in ischemic stroke.

**Methods:**

We comprehensively review the role of endothelial GLUT1 in ischemic stroke by narrating the findings concerning biological characteristics of GLUT1 in brain in depth, summarizing the changes of endothelial GLUT1 expression and activity during ischemic stroke, discussing how GLUT1 achieves its neuroprotective effect via maintaining endothelial function, and identifying some outstanding blind spots in current studies.

**Results:**

Endothelial GLUT1 maintains persistent high glucose and energy requirements of the brain by transporting glucose through the blood–brain barrier, which preserves endothelial function and is beneficial to stroke prognosis.

**Conclusion:**

This review underscores the potential involvement of GLUT1 trafficking, activity modulation, and degradation, and we look forward to more clinical and animal studies to illuminate these mechanisms.

## Graphical abstract

Following the endocytosis, internalized membrane‐bound vesicles containing GLUT1 protein enter the early endosome (EE) to start sorting process. In EE, selected GLUT1 cargoes can be directly recycled to the membrane, termed “fast recycling”, or interact with trans‐Golgi network, termed “trans‐Golgi transport”. Other GLUT1 cargoes then form the late endosome (LE) to either be degraded by the lysosome, termed “degradation” or be transported to the plasma membrane, termed “slow recycling”.

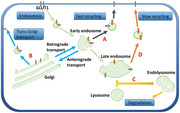

## 1 INTRODUCTION

Stroke is the second leading cause of disability and death worldwide (Collaborators GBDS, 2019). Currently, the only effective intervention to improve stroke outcomes is timely recanalization by intravenous thrombolysis or endovascular therapy (Phipps & Cronin, 2020). However, the overall effectiveness of these treatments is proved to be limited, with only 30%–50% of patients achieving good clinical long‐term functional outcome (Bi et al., 2021; Huo et al., 2023; Peng et al., 2022). Additionally, other interventions have proven largely ineffective in reducing the permanent neurological damage caused by stroke (Liu & Jia, 2023; Paul & Candelario‐Jalil, 2021), which highlights the urgent need for alternative treatments.

After the onset of the ischemic stroke, various pathological processes following energy depletion take place within minutes and sustain for hours or up to days, which ultimately exacerbates the damage in ischemia brain. The progression of stroke damage is mostly attributed to the dysfunction of blood–brain barrier (BBB). BBB is a highly differentiated brain structure composed of vascular endothelial cells (EC), pericytes, astrocyte endfeet, and basement membranes (Daneman & Prat, 2015). Among all the BBB components, ECs are the most vulnerable during the process of ischemia. ECs form cerebrovascular walls sealed with tight junctions (TJs), which highly restrict the movement of ions, molecules, and cells from blood to brain parenchyma in order to maintain a stable environment in brain. However, when ischemia occurs, EC permeability loosens in response to energy stress, leading to BBB leakage (Hu et al., 2017). In addition to their barrier function (Aird, 2004), ECs play a pivotal role in various BBB functions, including the transport of oxygen and nutrients, regulation of vasomotor tone, control of leukocyte and platelet adhesion and aggregation, secretion of angiocrine factors (Anggard, 1990; Rafii et al., 2016), and promotion of angiogenesis (Veys et al., 2020), all of which make ECs important to maintain central nervous system (CNS) homeostasis.

During ischemic stroke, the obstruction of blood glucose supply leads to serious energy crisis (Dirnagl et al., 1999), which causes EC dysfunction and therefore adds to the progression of stroke damage. ECs have high glycolytic activity and rely heavily on glucose for ATP production (De Bock et al., 2013). Insufficient glucose uptake significantly affects the function of ECs (Pi et al., 2018). The dominant glucose transporter (GLUT) in ECs is GLUT1, which determines not only the glucose flux into ECs, but also glucose transport across ECs into the brain parenchyma. GLUT1 deficiency due to gene mutations disturbs the glucose transport at the BBB and severely affects the development and function of CNS (De Vivo et al., 1991). Decreased GLUT1 at the BBB potentially causes the vascular pathologies prior to the onset of pathophysiological changes and symptoms of neurological diseases (Lyros et al., 2014; Zlokovic, 2011). Thus, it is important to investigate the role of GLUT1, which may contribute to preserving the function of ECs as well as conferring neuroprotection in ischemic stroke.

In this review, we thoroughly discuss the important protective role of EC‐GLUT1 after ischemic stroke. First, we review the distribution and physiological role of GLUT1. Then, to review the alteration of EC‐GLUT1 after stroke, we summarize our current knowledge on how ischemic stroke influences EC‐GLUT1 expression and activity. Next, we retrospect how changes in EC function lead to stroke damage and emphatically discuss how GLUT1 confers neuroprotection by maintaining EC function during stroke. Finally, we discuss some outstanding problems in current research studies in the hope of inspiring further investigation concerning the role of GLUT1 in stroke.

## 2 GLUT1 IS THE DOMINANT GLUCOSE TRANSPORTER IN BRAIN

**FIGURE 1:**
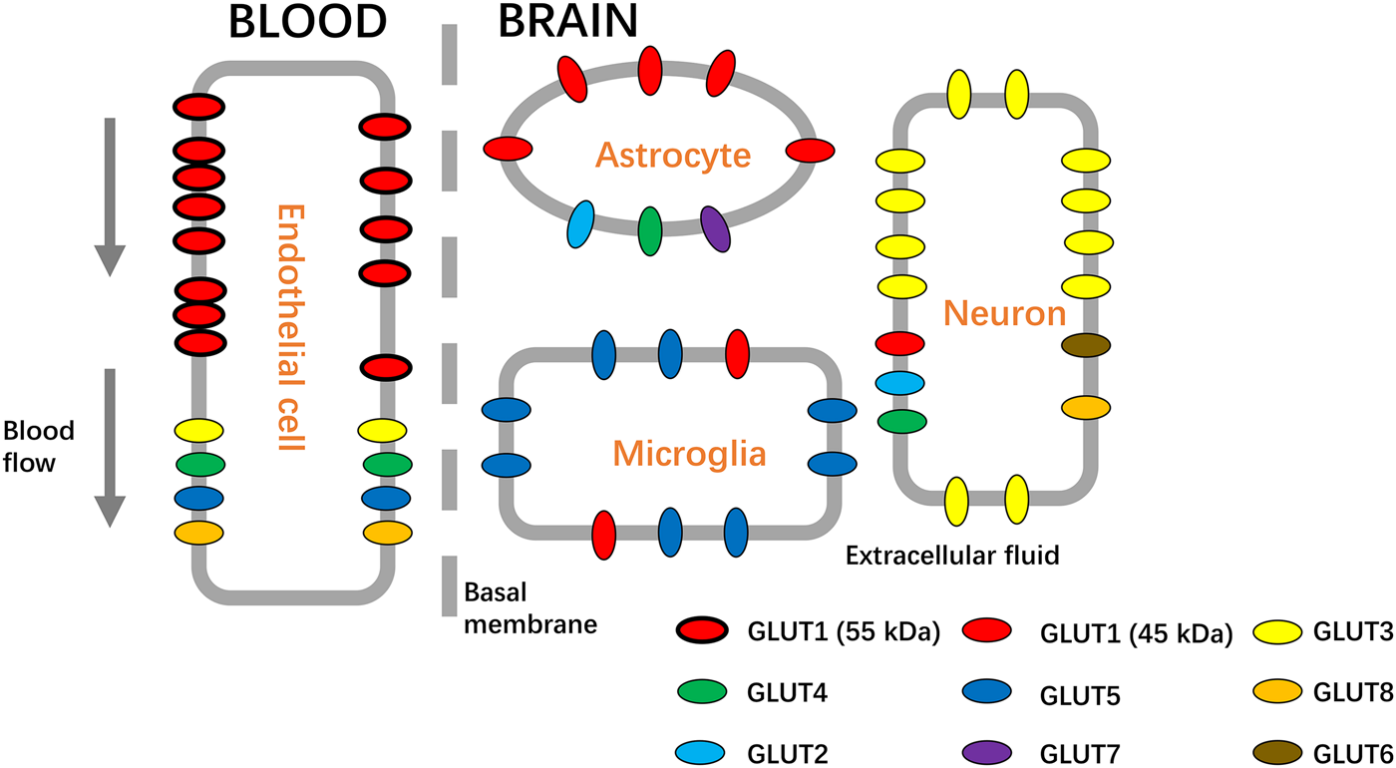
Glucose transporter (GLUT) distribution at the neurovascular unit (NVU). The most abundant glucose transporter in brain is GLUT1 (red), which is present in endothelial cells (ECs) in a highly glycosylated form of 55 kDa (thick border) and in astrocytes, microglia and neurons in a low glycosylated form of 45 kDa (thin border). In addition, the distributions of other glucose transporters at the NVU of the BBB are also shown: GLUT2 (pale blue), GLUT3 (yellow), GLUT4 (green), GLUT5 (blue), GLUT6 (brown), GLUT7 (purple), GLUT8 (orange).

To date, a total of 14 human GLUT proteins have been identified, among which GLUT1–6, 8, and 13 have been detected in brain (Mueckler & Thorens, 2013; Simpson et al., 2007). GLUT1, as the first recognized GLUT protein, expresses high level in the embryo period, the expression of which declines rapidly as gestation progresses. In addition, after birth, the expression of GLUT1 starts to increase gradually (Matsumoto et al., 1995). GLUT1 is abundantly expressed and widely distributed subtype among different tissues (Baldwin, 1993). Except for being expressed in erythrocyte membranes, GLUT1 is also highly expressed in the brain, eyes, peripheral nerves, kidney, colon, placental endothelium, as well as epithelial‐like barriers (Augustin, 2010; Kahn, 1992). In brain, the highest concentration of GLUT is GLUT1, which is distributed in ECs (mostly distributed), astrocytes, neurons, and microglia (Patching, 2017) (Figure 1).

GLUT1 is encoded from the SLC2A1 gene. It has been demonstrated that the embryonic lethality of homozygous SLC2A1^−/−^ knockout mice model (Wang et al., 2006), whereas the heterozygous SLC2A1^+/−^ mice model could mimic the features of people with GLUT1 deficiency syndrome, causing brain development delay, epilepsy, cognitive dysfunction, and so on (Benarroch, 2014). In addition, the weight of the brain is reduced in SLC2A1^+/−^ mice model, as is the glucose in the cerebrospinal fluid (Augustin, 2010). According to the studies above, we can draw the importance of GLUT1 for brain development. What is more, it is reported that SLC2A1^+/−^ mice present increased permeability of BBB owing to the lower TJs levels (Winkler et al., 2015). Lower GLUT1 protein levels on BBB result in delaying the angiogenesis of brain as well as triggering microvasculature diminution, though the BBB integrity remains intact (Tang et al., 2017). These findings suggest GLUT1 is essential to the endothelial function of maintaining BBB integrity and regular angiogenesis.

In the mature brain, GLUT1 has two molecular weight forms: 55 and 45 kDa. The former isoform has an additional *N*‐linked glycosylation site at Asn 45 on the first extracellular loop (Kumagai et al., 1994) and is expressed mainly in ECs, whereas the latter is expressed in cortical neuronal membranes and perivascular astrocyte endfeet (Roberts et al., 2008) (Figure 1, Zhang, Zuo, et al., 2014). GLUT1 distributes asymmetrically at the luminal (blood‐facing) and the abluminal (brain parenchyma–facing) membranes of the ECs (McAllister et al., 2001). In human, GLUT1 transporters in the luminal are three to four times more abundant than in the abluminal membrane (Cornford et al., 1994, 1998), whereas rodent brains show the opposite distribution pattern (Farrell & Pardridge, 1991). To explain this distribution, the transport of glucose molecules across the BBB is mainly achieved by GLUT1, and the glucose is rapidly catabolized in brain, creating a descending gradient for GLUT1 to transport glucose from the blood to the interstitial fluid of the brain. In addition, the distribution of GLUT1 in the luminal and abluminal membranes responds to the varying transmembrane concentration gradient of hexokinase (McAllister et al., 2001). Thus, changes in these ratios of the GLUT1 distribution seem to regulate the rate of glucose transport across the BBB in response to the varying energy demand of brain (Patching, 2017).

## 3 REGULATION OF EC‐GLUT1 AFTER STROKE

### 3.1 Alterations of EC‐GLUT1 after stroke

To fully comprehend the role of EC‐GLUT1 following stroke, the primary focus is on understanding the impact of stroke on the expression of GLUT1 in ECs. As one of the earliest pioneers, Vannucci et al. (1996) found that both damaged and undamaged hemispheres of the immature rat exhibited increased GLUT1 at 24 h after ischemia, whereas GLUT1 sustained elevated levels only in the ischemic hemisphere at 72 h after ischemia. Furthermore, the expression of GLUT1 in the brain is found to be elevated in both the microvascular and parenchymal regions at 24 h post‐ischemia (McCall et al., 1996). Specifically, the increase in GLUT1 expression appears to be primarily observed in ECs. In a rat model of transient middle cerebral artery occlusion (tMCAO), GLUT1 immunoreactivity is mainly collocated with anti‐rat EC antigen (RECA1) in the brain 24 h after ischemia (Iwata et al., 2014). Consistent with this finding, it is observed that the mRNA expression level of GLUT1 is significantly increased in ECs treated with oxygen and glucose deprivation and reperfusion (OGD/R) after a 48‐h reperfusion period (Tornabene et al., 2019). Given that GLUT1 serves as the primary GLUT in ECs, its upregulation following stroke could be viewed as an acute response to energy stress aimed at facilitating energy metabolism. Notably, no present study illustrates GLUT1 expression in brain beyond 72 h after ischemic stroke, which should be elucidated in future studies for a better understanding of GLUT1 function in stroke.

Although the expression of GLUT1 is elevated during stroke, the transport of glucose into ECs also depends on functional GLUT1. In the four‐vessel occlusion rat model, Suzuki et al. (1998) demonstrated that both maximum transport rate and the half saturation constant of the glucose transport were significantly decreased. These findings have brought to light that both the quantity of GLUT1 and its affinity for glucose experience a decline in the aftermath of stroke. These results reveal that both the number of functional GLUT1 and affinity of GLUT1 for glucose are decreased in response to stroke, which further indicates that the increased GLUT1 after ischemia are low in transport activity, in other words, malfunctioned. The malfunction of GLUT1 in stroke models raises new questions for discussion. First, what mechanisms lead to the function impairment of GLUT1 in the context of stroke? Second, does the compromised performance of GLUT1 exacerbate the detrimental effects of stroke? Third, is it plausible that the restoration of GLUT1 function could potentially enhance the outcome of a stroke? These inquiries hold significant importance in comprehending the intricate role of GLUT1 in the advancement of stroke. Subsequently, we aim to delve deeply into the subsequent sections, thoroughly exploring the regulation, function, and potential neuroprotective contributions of GLUT1 post‐stroke.

### 3.2 Mechanism of EC‐GLUT1 regulation after stroke

In this section, we review GLUT1 regulation during ischemic stroke from the expression and activity of GLUT1 in ECs. Our review encompasses several vital aspects, spanning *GLUT1* gene transcription, mRNA stability, and potential mechanisms that govern protein activity. These mechanisms encompass protein structural modifications, the translocation of GLUT1 from the cytoplasm to the cell membrane, and the potential inadequate degradation of dysfunctional GLUT1. These alterations play crucial role in the regulation of GLUT1 and might be key to reversing the impaired GLUT1 activity in ischemic stroke.

#### 3.2.1 *GLUT1* gene transcription

Transcriptional factors activated by glucose deprivation or hypoxia regulate EC‐GLUT1 expression by binding to *cis*‐acting regulatory elements in the 3′ untranslated region (UTR) of the *GLUT1* gene (Gantt et al., 2006; Hamilton et al., 1999). The core promoter of *GLUT1* gene is in the −99/−33 region in rats, and the GC box at −91/−86 and TATA box within the promoter are indispensable for *GLUT1* transcription activity (Murakami et al., 1992; Sanchez‐Feutrie et al., 2004; Santalucia et al., 2005).

Hypoxia‐inducible factor 1α (HIF‐1α) is a well‐established transcriptional factor to upregulate GLUT1 after stroke (Jones et al., 2018). HIF‐1α is rapidly expressed and activated following ischemic stroke (Yang et al., 2017) and critically mediates energy metabolism homeostasis under hypoxia conditions by promoting gene transcription. *GLUT1* promoter region contains a 184 bp hypoxia‐responsive element (HRE), which is located at about −3.2/−3.0 kb from the transcription start site. HIF‐1α can directly bind to HRE to increase *GLUT1* gene expression under low‐oxygen conditions (Hayashi et al., 2004), and HIF‐1α siRNA significantly eliminated hypoxic‐induced GLUT1 expression in mammalian ECs (Shao et al., 2014). HIF‐1α may also be critical in mediating endothelial response to cellular crosstalk, for HIF‐1α siRNA transfection to ECs can abrogate GLUT1 expression in ECs induced by hypoxic astrocytes (Choi, 2017).

Several other transcription factors, including NF‐κB, SIRT1, forkhead box protein M1, and tensin homolog on chromosome 10, have also been revealed to regulate GLUT1 expression (Chen et al., 2019; Phadngam et al., 2016; Wang et al., 2016) and participate in ischemic stroke, but their possible roles of regulating EC‐GLUT1 during stroke require further demonstration.

Aside from activation of transcription factors, histone modification is also common in stroke and also plays an important part in controlling *GLUT1* gene transcription (Kao & Lin, 2019). Inhibition of histone deacetylase (HDAC) leads to significant *GLUT1* acetylation and increases GLUT1 expression (Chen et al., 2015). In fasted mice, the critical *cis*‐regulatory region in *GLUT1* gene shows H3K9 hyperacetylation in ECs, accompanied with upregulated GLUT1 expression (Tanegashima et al., 2017), which postulates that histone modification responds to energy deprivation. Therefore, it is also worthy of investigation whether this mechanism also initiated the EC‐GLUT1 expression change in ischemic stroke.

#### 3.2.2 GLUT1 mRNA stability

When exposed to glucose deprivation, the half‐life of GLUT1 mRNA is increased in brain ECs (Boado & Pardridge, 1993), which indicates that increased mRNA stability also contributes to upregulation of GLUT1 expression in stroke.

The 3′‐UTR of GLUT1 mRNA contains a 10 nt *cis*‐acting element that is rich in adenosine–uridine (A–U) (Boado & Pardridge, 1998; Dwyer et al., 1996). The A–U‐rich element (ARE) can interact with a variety of A–U binding proteins to stabilize or destabilize GLUT1 mRNA according to different cell states (Bohjanen et al., 1991; Gaugitsch et al., 1992; Malter, 1989; Vakalopoulou et al., 1991). HuR protein, a widely expressed RNA‐binding protein, is one of the A–U binding proteins, and RNA gel shift analysis suggests it can target ARE in the GLUT1 3′‐UTR (Gantt et al., 2006). siRNA‐mediated reduction of HuR protein results in downregulation of GLUT1 protein expression (Gantt et al., 2006), which indicates a requirement for the HuR‐GLUT1 mRNA complex to maintain GLUT1 expression. Interestingly, HuR has been found to regulate several ARE‐containing mRNAs in cytoplasm during global cerebral ischemia (Jamison et al., 2008), suggesting that HuR may also be involved in regulating GLUT1 expression in stroke.

The cytokine tumor necrosis factor‐α can also increase the stability of GLUT1 mRNA by interacting with GLUT1 UTR (McGowan et al., 1997), which is likely to occur during ischemic stroke (Billinger et al., 2017). Brain‐derived peptide cerebrolysin, a neuroprotector in stroke (Catalin et al., 2018), increases GLUT1 transcript stability in BBB as well as glucose uptake in ECs (Boado, 1995, 2001). On the other hand, when exposed to high‐glucose conditions, calreticulin is activated in vascular ECs to decrease mRNA stability (Totary‐Jain et al., 2005). Such regulation mechanism may be of importance for stroke patients with diabetes and might partly contribute to their vulnerability to stroke damage.

#### 3.2.3 GLUT1 activity

The activity of GLUT1 is fundamental to ensure sufficient glucose transport into brain, and impaired kinetics of GLUT1 in ischemic stroke have been implied. Internal disulfide bond connection formation within GLUT1 has shown to enhance GLUT1 activity (Carruthers et al., 2009; Zottola et al., 1995). It is worth mentioning that nitroxyl, the product of a one‐electron reduction of NO, is demonstrated to improve GLUT1 activity by promoting internal disulfide bond formation (Salie et al., 2012). Moreover, vascular ECs are able to produce endogenous nitroxyl, which has vasodilator actions (Tare et al., 2017), and pharmacological treatment with nitroxyl donors to stroke survivals shows promising protective effect in a series of clinical trials (Miranda et al., 2005). These results suggest that upregulating nitroxyl in ECs could be a potential target to improve GLUT1 activity and confer neuroprotection in ischemic stroke.

Interestingly, adenosine and ATP are found to bind with GLUT1 in vitro and influence the substrate‐binding affinity of GLUT1: adenosine decreases its glucose affinity, whereas ATP increases this affinity (Lachaal et al., 2001). As ischemia increases adenosine/ATP ratio in the infarcted brain due to acute energy deprivation, this imbalance may produce suppressive effect on GLUT1 activity and cause a vicious cascade that worsens energy deficiency in stroke. Similarly, alkaline condition is demonstrated to favor GLUT1 transport activation (Gunnink et al., 2014). During stroke, deprivation of oxygen often causes decreased pH in brain parenchyma, which represents the acidity of environment and lowers GLUT1 activity (Pochechuev et al., 2022; Shah et al., 2015). To conclude, the increased adenosine/ATP ratio or decreased pH may suppress GLUT1 activity during stroke, which is likely to exacerbate the energy deficiency and lead to further damage.

Previous study has also indicated the important role of lipid rafts in suppressing sporadic cluster formation to inhibit GLUT1 activity (Zheng et al., 2018). Glucose deprivation can induce GLUT1 to bind to the membrane protein stomatin, which in turn facilitates the interaction between GLUT1 and lipid rafts (Kumar et al., 2004). Overexpressing stomatin in a mixed population of stably transfected Clone 9 cells reduces its glucose uptake, indicating decreased GLUT1 activity (Zhang et al., 2001). These studies indicate that decreased binding of GLUT1 with stomatin may also contribute to lower GLUT1 activity in ischemic ECs.

#### 3.2.4 GLUT1 trafficking

**FIGURE 2:**
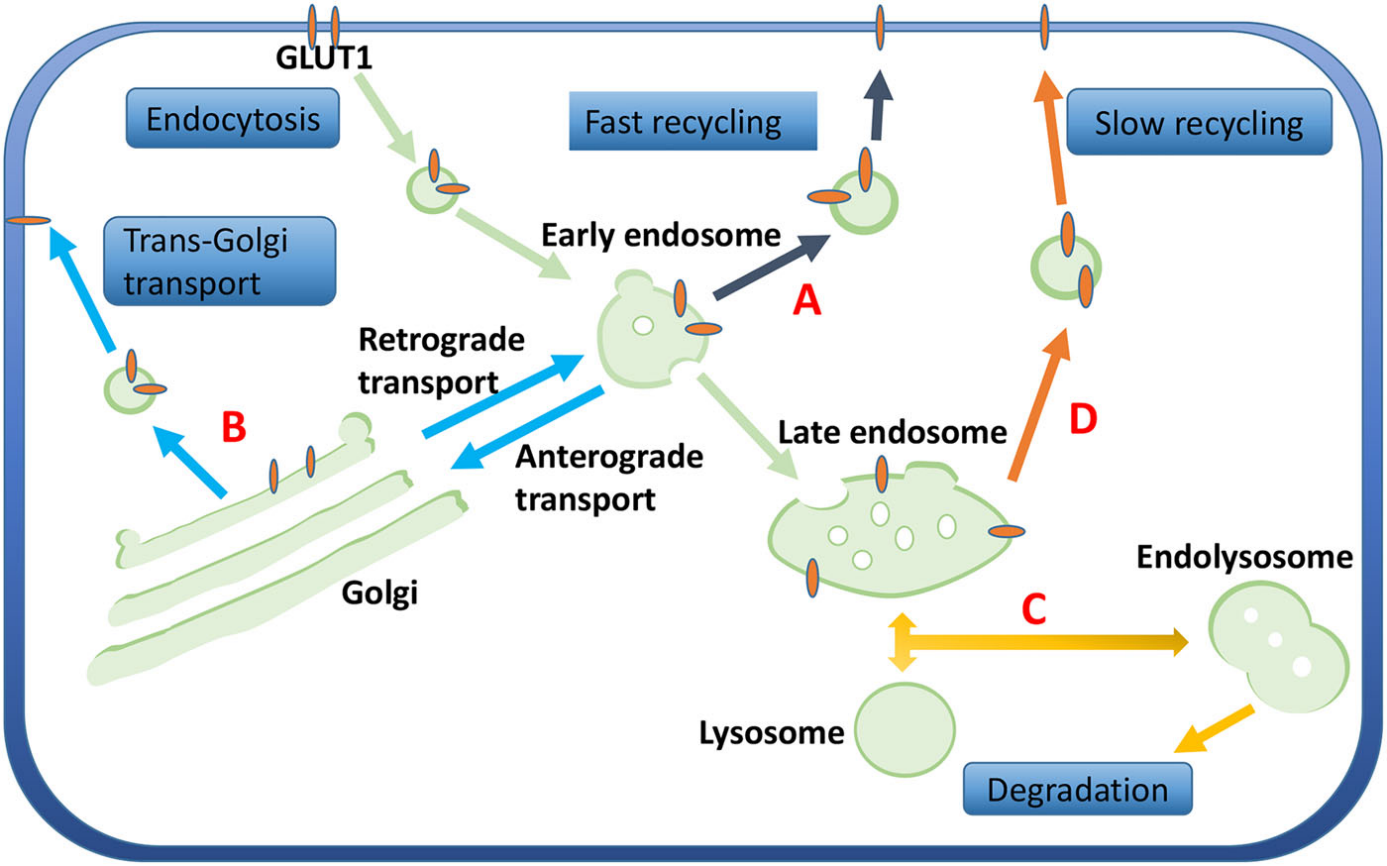
The overview of glucose transporter 1 (GLUT1) trafficking. Following the endocytosis, internalized membrane‐bound vesicles containing GLUT1 protein enter the early endosome (EE) to start its sorting process. In EE, selected GLUT1 cargoes can (A) be directly recycled to the membrane, termed “fast recycling,” or (B) interact with trans‐Golgi network through anterograde and retrograde transport and target back to the cell surface, termed “trans‐Golgi transport.” Other GLUT1 cargoes then form the late endosome (LE) to either (C) be degraded by the lysosome, termed “degradation,” or (D) be transported to the plasma membrane, termed “slow recycling.”

The basic maintenance of GLUT1 on cell membrane involves a dynamic process: cell membrane GLUT1 is internalized into cytoplasm through endocytosis, which refers to the membranes budding from plasma membrane lipid bilayer into cytoplasm (Wieman et al., 2007). Internalized cytoplasmic GLUT1 can be either degraded by autophagy, involving the participation of lysosome (Edinger et al., 2003), or recruited back to the plasma membrane to sustain surface GLUT1 level with the help of endosomal system and several key GLUT1 trafficking proteins (Steinberg et al., 2013; Wu et al., 2013); this process is defined as GLUT1 trafficking (Figure 2).

It is worth noting that GLUT1 trafficking is also a common and essential process in ischemic situations. Young et al. (1997) first revealed that GLUT1 can be translocated from intracellular storage pool to sarcolemma to increase glucose uptake in ischemic myocardial cells, and hyperinsulinemia seemed to have an additive effect on GLUT1 trafficking (Russell et al., 1998). GLUT1 translocation to membrane significantly improves the myocardial uptake of [^18^F] fluorodeoxyglucose (FDG) and alleviates energy crises during ischemia, which underlines the significance of GLUT1 membrane trafficking in ischemic condition. In the OGD/R model of primary bovine ECs, Erica et al. used immunolabeling and confocal laser microscopy analysis to detect the translocation of GLUT1 and found that it was mainly localized in the cytosol 4 h after OGD, whereas the concentration at cell border was increased 24 h after reperfusion (Tornabene et al., 2019). However, the underlining mechanism regulating GLUT1 translocation during stroke remains elusive. We summarize evidence of GLUT1 trafficking under pathological conditions in the following sections and hope to offer insights into GLUT1 translocation mechanism in ischemic stroke.

Thioredoxin‐interacting protein (TXNIP), an α‐arrestin family protein, has been found to interact with GLUT1 and inhibit its ability to transport glucose at the plasma membrane through promoting GLUT1 endocytosis. Energy stress can activate AMP‐dependent protein kinase to phosphorylate TXNIP, which in turn induces TXNIP degradation. These subsequently compromise TXNIP‐mediated GLUT1 endocytosis and increase glucose uptake (Wu et al., 2013). Of note, TXNIP is highly expressed in brain ECs (Zhang, Chen, et al., 2014) and regulates many substantial endothelial functions, such as angiogenesis, autoxidation, and alignment (Devi et al., 2012; Park et al., 2013; Spindel et al., 2014). The suppression of TXNIP/NLRP3 inflammasome can abrogate endothelial dysfunction, conferring neuroprotection in an ischemic stroke model (Cao et al., 2016). These findings highlight the vital role of TXNIP in regulating endothelial functions. Considering both endothelial dysfunction and GLUT1 dysregulation are common in ischemic stroke and TXNIP can influence both changes, it can be assumed that TXNIP may be a potential target to regulate GLUT1 in ischemic ECs.

Autophagy also plays a crucial role in protein recycling and degradation. Equivocal evidence indicates that GLUT1 trafficking is critically influenced by autophagy. In the OGD/R model of ECs, the ratio of LC3‐II‐to‐LC3‐I protein is significantly increased, which indicates the activation of autophagy (Kim et al., 2018; Zhang et al., 2018). Moreover, increased GLUT1 translocation has been found in OGD/R treated ECs (Tornabene et al., 2019). Recently, Roy et al. (2017) found that GLUT1 was predominantly located with late endosomes, and the cell surface GLUT1 was decreased in *trans*‐genetic autophagy‐deficient cells when exposed to hypoxia. Mechanically, LC3 can target TBC1D5 (also denoted AS160, which can inhibit GLUT1 membrane trafficking) to induce its degradation under hypoxic condition, which will then increase the retromer complex activity and promote GLUT1 trafficking to cell surface membrane (Roy et al., 2017).

#### 3.2.5 GLUT1 degradation

The content of GLUT1 is in a state of dynamic equilibrium. Cells synthesize new functional GLUT1 and degrade nonfunctional GLUT1 to maintain the activity and expression of GLUT1 at a stable level. Impaired degradation process may lead to the accumulation of nonfunctional GLUT1 and aggravate energy deficits. Meanwhile, exacerbated degradation could also lead to loss of available GLUT1 during ischemic stroke.

Ischemic stroke has been observed to trigger excessive ubiquitination and degradation of proteins in cerebral ECs, resulting in the disruption of cell‐to‐cell junctions and subsequently raising vascular permeability (Sewduth et al., 2017). In retinal ECs cultured with high glucose concentration, ubiquitin increasingly binds to GLUT1, and increased ubiquitination of GLUT1 promotes its degradation (Fernandes et al., 2004). These findings suggest the presence of ubiquitin‐dependent system in the degradation of GLUT1. Therefore, ubiquitination might also play a role in reducing GLUT1 activity by facilitating its degradation in brain ischemia.

The SUMOylation of GLUT1 promotes its degradation, which interferes with glucose transport (Giorgino et al., 2000). Ischemia can also elevate the levels of SUMOylated proteins in the brain, which is likely to result in the degradation of GLUT1 as well (Yang et al., 2008). Furthermore, SUMOylation enhances autophagy, and robust autophagic degradation necessitates the SUMOylation of parkin (Um & Chung, 2006). Compared with the existence of protein SUMOylation in ischemia condition, GLUT1 SUMOylation is more likely to promote the GLUT1 degradation process during stroke.

## 4 THE EFFECT OF EC‐GLUT1 ON NEUROPROTECTION AFTER ISCHEMIC STROKE

GLUT1 assumes a pivotal role in the context of stroke due to its crucial involvement in maintaining EC energy equilibrium. Furthermore, compromised EC function significantly impacts the advancement of stroke‐induced damage. In this section, we will review the neuroprotection of GLUT1 during stroke by emphasizing its effect in maintaining EC function.

### 4.1 GLUT1 is critical for protecting ECs in stroke

Ischemic stroke triggers pathological alterations in brain ECs, leading to EC dysfunction, which detrimentally impacts the prognosis of stroke. GLUT1 is intricately linked with the ischemia‐induced energy insufficiency and oxidative stress in brain ECs. Consequently, our initial emphasis is on highlighting the pivotal role of GLUT1 in safeguarding ECs from damage. This is achieved by discussing how GLUT1 engages in vital processes that contribute to the preservation of EC integrity during stroke.

In the event of blood supply disruption, cerebral ECs experience an energy collapse (Nguyen et al., 2018). The inhibition of EC‐GLUT1 expression has been observed to decrease the synthesis of intracellular ATP and lactate. Conversely, increased expression of GLUT1 can result in heightened ATP production in ECs (Hu et al., 2019; Wang et al., 2018). Similarly, GLUT1 expression induced by pre‐treatment with progesterone compensates energy demand and confers neuroprotection in ischemic stroke (Li et al., 2013). These findings therefore underline the significant role of GLUT1 in sustaining EC metabolism during ischemic stroke.

Another notable pathological alteration in acute ischemic stroke is the excessive generation of reactive oxygen radicals (ROS), which stems from heightened Ca^2+^ influx, excitotoxicity, and mitochondrial dysfunction (Lafon‐Cazal et al., 1993; Piantadosi & Zhang, 1996). Specifically, within cerebral ECs, ischemia can trigger an overproduction of superoxide anions. These anions subsequently combine with nitric oxide (NO) to form peroxynitrite, thereby instigating EC damage and apoptosis (Heo et al., 2005; Salvemini et al., 2006). Therefore, reducing ROS is crucial in enhancing the viability of EC and counteracting EC dysfunction induced by oxidative stress (Cai et al., 2018), where GLUT1 plays a pivotal role in the modulation of ROS within ECs. The inhibition of GLUT1 leads to an elevation in ROS levels (Andrisse et al., 2014), implying that GLUT1 could potentially safeguard ECs against oxidative damage, particularly in situations like ischemic stroke where GLUT1 expression is frequently upregulated. In addition to providing energy, glucose can also be phosphorylated to form glucose‐6‐phosphate, which subsequently enters the pentose phosphate pathway, serving as the primary source of nicotinamide adenine dinucleotide phosphate (NADPH) (Kruger & von Schaewen, 2003). NADPH functions as the primary scavenger of intracellular ROS and contributes electrons to facilitate antioxidant pathways (Hanukoglu & Rapoport, 1995).

Vitamin C serves as an additional scavenger for ROS within the CNS, and its transportation relies on both the sodium‐dependent vitamin C transporter 2 and GLUT1. Interestingly, GLUT1 has been identified as a transporter for the oxidized form of vitamin C, thereby participating in the regulation of oxidative stress within the brain (Linster et al., 2007). Daily intake of vitamin C markedly increases the expression of GLUT1 in ECs, and this in turn facilitates the recruitment of vitamin C, attenuating the ROS generation in the ischemic penumbra (Iwata et al., 2014).

GLUT1 also transports dehydroascorbate (DHA), which ultimately accumulates in the mitochondria to quench ROS, thus protecting mitochondria from oxidative injury (Kc et al., 2005). It suggests that DHA uptake via GLUT1 may protect ECs from ROS in ischemic stroke.

### 4.2 The involvement of EC‐GLUT1 in stroke pathology

The occurrence of ischemic injury results in a range of pathological changes in brain, including disruptions in BBB, irregularities in substance transport, disturbed blood flow, and abnormal angiogenesis, collectively influencing the prognosis of stroke. Interestingly, EC‐GLUT1 plays a crucial role in modulating diverse aspects of EC function, encompassing the regulation of BBB permeability, substance transport, vascular tension, and angiogenesis. This underscores its significance as a crucial target in the context of cerebral ischemia.

#### 4.2.1 BBB disruption

In physiological conditions, the permeability of BBB is sustained by the highly modulated *trans*‐ and paracellular regulations of ECs, maintaining the stable substance transportation. However, ischemic stimuli can swiftly induce EC dysfunction, subsequently resulting in BBB breakdown. This breakdown is characterized by the excessive and unselective entry of vascular components into the brain parenchyma (Candelario‐Jalil et al., 2022). In stroke patients, BBB disruption is evident in magnetic resonance imaging by contrast enhancement on T1‐weighted imaging just 3 h after the stroke event (Giraud et al., 2015). In a mice model of tMCAO, the BBB leakage confirmed by Evan's blue‐albumin in infarcted parenchyma is detected as early as 6 h after reperfusion (Li et al., 2017). As highlighted earlier, EC‐GLUT1 holds a critical role in sustaining EC function, and numerous studies have substantiated that EC‐GLUT1 can help preserve BBB integrity during ischemic stroke (Lee et al., 2022; Veys et al., 2020; Zheng et al., 2010).

In the brain of adult *trans*‐genetic SLC2A1^+/−^ mice, the microvascular leakage of endogenous plasma proteins (including fibrin and immunoglobulin G) is increased by around 10‐fold compared to SLC2A1^+/+^ littermates. Moreover, there is a significant decrease in the expression of TJs proteins such as occludin and zonula occludens‐1 (ZO‐1) in the brains of SLC2A1^+/−^ mice (Winkler et al., 2015). This indicates the significant importance of GLUT1 in maintaining the BBB.

In in vitro BBB models established by primary human brain ECs, the treatment with methamphetamine (METH), a drug known to impair the expression of GLUT1 in ECs, leads to decreased *trans*‐endothelial electrical resistance (TEER) and increased accumulation of both small and large molecular weight tracer across the BBB. These changes indicate elevated BBB leakage. Similarly, following METH‐induced reduction of GLUT1 expression, there is a decrease observed in the expression of BBB TJ proteins, including occluding and ZO‐1 (Abdul Muneer et al., 2011). These findings suggest that an insufficient level of GLUT1 protein can lead to BBB disruption.

Interestingly, insufficient GLUT1 level seems to affect the integrity of BBB only when occurring in ECs, for conditional knockout of GLUT1 in ECs rather than in astrocyte‐induced BBB disrupted phenotype (Winkler et al., 2015), which suggests that the crucial EC function for maintaining intact BBB is dependent on GLUT1 expression. These findings are particularly relevant, as cerebral EC‐GLUT1 expression after ischemic stroke appears to be closely linked to stroke outcomes. Thus, increasing EC‐GLUT1 expression might improve stroke prognosis by alleviating BBB breakdown.

In the tMCAO rat model, increased GLUT1 levels alleviate Evan's blue dye leakage and therefore prevent the BBB disruption (Ha Park et al., 2016). Additionally, diabetic tMCAO rats exhibit lower cerebral EC‐GLUT1 level after ischemic stroke, along with intensified cerebral edema and increased vulnerability to ischemic stroke damage (Iwata et al., 2014). In alignment with this, upregulating EC‐GLUT1 alleviates BBB leakage and reduces brain edema in diabetic tMCAO rats (Xia et al., 2018). These results might partly explain why diabetes mellitus (DM) leads to poorer outcome in stroke patients (Lau et al., 2019), as an inadequate level of GLUT1 exacerbates BBB damage, and provide insights into the neuroprotection of stroke patients with DM.

#### 4.2.2 Substance transportation abnormality

Another hallmark of stroke progression is the disrupted transport of substances across the endothelial barrier into the brain. Being an essential transporter, EC‐GLUT1 has been identified as the key regulator of both glucose and DHA transport within the CNS. Moreover, evidence has demonstrated the fundamental role of EC‐GLUT1 in maintaining proper substance transport in the context of stroke.

After ischemic stroke, there is a significant decrease in glucose uptake in the ischemic region (Deng et al., 2018), indicating a reduction in glucose metabolism during ischemic stroke. Treatments that augment EC‐GLUT1 expression have been shown to bolster glucose uptake and safeguard against brain EC loss in the rat tMCAO model, highlighting its pivotal role in energy supply (Shi et al., 1997).

Besides glucose transport, the concentration of DHA within brain tissue is also significantly reduced during cerebral ischemia (Mack et al., 2006), and this decline is attributed to compromised EC DHA transportation. In the context of ischemic stroke, vitamin C serves as a critical antioxidant that counteracts ROS, and administering vitamin C prior to ischemia has been shown to reduce infarct volume and confer neuroprotective effects in rat models of ischemic stroke (Li et al., 2019).

Mechanically, studies have revealed that DHA can enter the mitochondria through GLUT1 to counteract mitochondrial ROS and therefore protect mitochondrial DNA from oxidative damage in 293T cells (Kc et al., 2005). Furthermore, in the rat tMCAO model, vitamin C supplement upregulates GLUT1 expression and diminishes the production of superoxide radical in the ischemic penumbra (Iwata et al., 2014). Based on these findings, it can be inferred that GLUT1 plays a role in transporting DHA during ischemic stroke. Given the heightened oxidative environment post‐cerebral infarction, ECs‐GLUT1‐mediated DHA transport assumes a significant role in alleviating oxidative stress and offering neuroprotection.

#### 4.2.3 Dysregulated blood flow

There is substantial evidence that ischemic stroke can lead to microcirculatory disturbance in brain, regardless of recanalization occurs. In response to the dynamic oxygen and metabolic demands of brain, ECs can modulate vascular tension to regulate blood flow, primarily achieved through the synthesis and release of nitric oxide (Palmer et al., 1987). Intriguingly, it has been reported that EC‐GLUT1 has an impact on NO synthesis, thereby contributing to the regulation of blood flow after ischemic stroke.

Importantly, the influence of EC‐GLUT1 on NO production in ECs has been well established. Inhibition of GLUT1 has been shown to suppress shear‐induced NO generation in ECs, and the overexpression of EC‐GLUT1 can counteract this inhibition (Bharath et al., 2017). Similarly, in GLUT1‐deficient transgenic mice, ECs exhibit impaired NO synthesis, and endothelium‐dependent relaxation is compromised (Park et al., 2004). This finding suggests that GLUT1 plays a crucial role in inducing NO synthesis within ECs, consequently promoting vasodilation.

Hypertension is known to alter shear stress and EC function, and hypertensive patients are often associated with poor prognosis after cerebral ischemic events (Cipolla et al., 2018). In stroke‐prone spontaneously hypertensive rats, GLUT1 expression is found to be lower than that in the control group after ischemic stroke (Ishida et al., 2006). It would be interesting to investigate whether insufficient GLUT1 expression also contributes to the impaired shear‐induced NO generation in hypertensive stroke patients, thus further aggravating poor prognosis.

#### 4.2.4 Angiogenesis

When blood supply is suspended, brain ECs can stimulate angiogenesis, a process that involves the restructuring of the vascular network and typically begins within 4–7 days after stroke (Kanazawa et al., 2017). Vascular endothelial growth factors (VEGFs), particularly VEGF‐A, play an important role in brain angiogenesis. Under ischemic conditions, ECs increase the secretion of VEGF, which then binds to VEGFR‐2 on endothelial progenitor cells (Carmeliet & Tessier‐Lavigne, 2005; Plate, 1999). This activation prompts endothelial progenitor cells to migrate to the damaged endothelium and extend toward the ischemic area, ultimately leading to the formation of new blood vessels (Esquiva et al., 2018; Liu et al., 2018). Significantly, angiogenesis after stroke has been reported to contribute to both neuronal remodeling and functional recovery in stroke patients.

EC‐GLUT1 appears to play a role in angiogenesis by regulating VEGF secretion following stroke. In diabetic mice, the increased expression of GLUT1 through HDAC inhibition has been linked to angiogenesis in the myocardium (Chen et al., 2015). More recently, in the mice model of GLUT1 deficiency syndrome, the brain's capillary network is significantly reduced and appeared fragmented. However, when these mutant mice are treated with AAV9‐GLUT1 virus, the restoration of GLUT1 leads to the development of an elaborate and dense microvasculature in brain. This is evidenced through both in vitro immunohistochemistry and in vivo two‐photon microscopy, highlighting that GLUT1 upregulation reinstated angiogenesis (Tang et al., 2017). Therefore, these experiments offer direct support for the involvement of GLUT1 in angiogenesis.

Furthermore, emerging evidence has indicated a dual interaction between the expression of GLUT1 and VEGF (Schuler et al., 2018). When exposed to hypoxic conditions, gliomas are capable of producing VEGF to enhance glucose uptake in the BBB, which is probably mediated by upregulated GLUT1 in ECs (Yeh et al., 2008). Conversely, specifically induced downregulation of EC‐GLUT1 expression leads to a decrease in serum VEGF concentration (Jais et al., 2016). Given that both angiogenesis and changes in GLUT1 expression are common occurrences in ischemic stroke and considering their close association, these findings suggest the potential role of GLUT1 in the regulation of VEGF and consequently the promotion of angiogenesis following stroke.

## 5 DISCUSSION

Energy crisis is a significant hallmark of cerebral ischemia, which arises due to the lack of oxygen and glucose supply in the infarcted brain region. GLUT1 is prominently expressed in ECs of BBB and is fundamental for continuous glucose supply to the brain. In the penumbra, a region with sustained yet reduced blood flow surrounding the infarct, there is a possibility that enhancing GLUT1 expression in this area could be advantageous. This enhancement could potentially facilitate the uptake of glucose by brain cells, subsequently promoting the distribution of glucose to the ischemic region. As a result, modulating EC‐GLUT1 within the BBB stands as a prospective approach for enhancing the prognosis of stroke.

Indeed, several studies have demonstrated the neuroprotective effect of GLUT1 upregulation in ischemic ECs. Progesterone and estrogen are found to increase the expression of GLUT1 in ECs, leading to improved stroke outcomes (Shi et al., 1997). Conversely, smoking preconditioning appears to hinder stroke recovery by compromising the ability to elevate GLUT1 expression in ischemia brain (Shah et al., 2015). However, there are still significant gaps in our understanding of the alterations and modulation of EC‐GLUT1, particularly within the penumbra area during cerebral ischemia. Presently, most studies have focused on broader parenchymal regions (Shi et al., 1997), and specific changes in the penumbra remain elusive.

Furthermore, existing research has primarily examined GLUT1 alterations up to 72 h post‐ischemia (Shi et al., 1997), marking a cutoff in the latent phase. Considering the prolonged rehabilitation required for stroke survivors, a longer time frame should be explored to offer insights for further investigations. Lastly, the mechanisms underlying changes in EC‐GLUT1 expression during stroke are only partially understood, with much of the current knowledge derived from studies involving tumors and DM. Consequently, more extensive research is warranted to elucidate the regulatory mechanisms of GLUT1 in ischemic stroke models.

In the future, confirming the neuroprotective effect of GLUT1 in stroke remains essential, as this would elucidate the potential therapeutic roles of GLUT1. Considering that stroke triggers an acute response in brain, it is plausible that GLUT1 translocation from the cytoplasm to the membrane plays a pivotal role in swiftly maintaining GLUT1 membrane localization in ECs (Leto & Saltiel, 2012). Moreover, attention should be directed toward the decrease in GLUT1 activity during stroke, as investigations into the structural and post‐translational modifications of GLUT1 have made significant strides in both basic and translational studies. Additionally, the phenomenon of GLUT1 degradation, commonly observed in the regulation of GLUT1, provides further insights into its role in stroke. Autophagic pathways might be integral to this process due to their crucial role in protein degradation.

In conclusion, this review underscores the potential involvement of GLUT1 trafficking, activity modulation, and degradation, and looks forward to more clinical and animal studies to illuminate these mechanisms.

## AUTHOR CONTRIBUTIONS

Qiwei Peng wrote the article; Weiqi Zeng reviewed and made corrections to the article.

## CONFLICT OF INTEREST STATEMENT

The authors declare that they have no conflicts of interest.

### PEER REVIEW

The peer review history for this article is available at https://publons.com/publon/10.1002/brb3.3536.

## Data Availability

Data availability is not applicable to this article as no new data were created or analyzed in this study.
